# Identification of important genes related to HVSMC proliferation and migration in graft restenosis based on WGCNA

**DOI:** 10.1038/s41598-024-51564-z

**Published:** 2024-01-12

**Authors:** Xiankun Liu, Mingzhen Qin, Qingliang Chen, Nan Jiang, Lianqun Wang, Yunpeng Bai, Zhigang Guo

**Affiliations:** 1 Clinical School of Thoracic, Tianjin Medical University, Tianjin, China; 2 Tianjin Chest Hospital, Tianjin Medical University, Tianjin, China; 3 Department of Cardiac Surgery, Chest Hospital, Tianjin University, Tianjin, China; 4 Tianjin Key Laboratory of Cardiovascular Emergency and Critical Care, Tianjin Municipal Science and Technology Bureau, Tianjin, China

**Keywords:** Data mining, Restenosis

## Abstract

The great saphenous vein is the most commonly used vessel for coronary artery bypass grafting (CABG), but its use has been associated with a high restenosis rate at 10-year follow-up. This study sought to determine the key genes associated with vein graft restenosis that could serve as novel therapeutic targets. A total of 3075 upregulated and 1404 downregulated genes were identified after transcriptome sequencing of three pairs of restenosed vein grafts and intraoperative spare great saphenous veins. Weighted gene co-expression network analysis showed that the floralwhite module had the highest correlation with vein graft restenosis. The intersection of the floralwhite module gene set and the upregulated gene set contained 615 upregulated genes strongly correlated with vein graft restenosis. Protein–protein interaction network analysis identified six hub genes (ITGAM, PTPRC, TLR4, TYROBP, ITGB2 and CD4), which were obtained using the STRING database and CytoHubba. Gene Ontology term and Kyoto Encyclopedia of Genes and Genomes pathway enrichment analyses showed that the common hub genes were mainly involved in the composition of the cell membrane; in biological processes such as neutrophil degranulation, receptor binding and intercellular adhesion, innate immune deficiency; and other signaling pathways. Finally, ITGB2 was selected as the target gene, and its expression was verified in tissues. The results showed that ITGB2 was significantly overexpressed in occluded vein grafts. To study the function of ITGB2 in HVSMCs, primary HVSMCs were cultured and successfully identified. EdU incorporation, wound healing and transwell assays showed that ITGB2 silencing significantly inhibited the proliferation and migration of HVSMCs stimulated by PDGF-BB. Overall, our study provides a basis for future studies on preventing restenosis following CABG.

## Introduction

Coronary artery bypass grafting (CABG) remains the gold standard for treating patients with complex multivessel coronary artery disease and/or left main disease, diabetes or reduced left ventricular function, according to US and European guidelines^1,2^. Saphenous vein grafts (SVGs) are the most frequently used conduits for CABG, but their use is associated with a 10-year vein graft restenosis rate of 40–50%^3^. However, the mechanism of vein graft restenosis remains unclear, although it is widely thought to be related to multiple factors and mechanisms that cause intimal hyperplasia (IH), mainly involving the proliferation and migration of human vein smooth muscle cells (HVSMCs). Therefore, it is essential to further study the molecular mechanisms of IH following CABG.

Bioinformatics is a discipline based on integration of life sciences and computer sciences that involves the collection, processing, storage, transmission, analysis and interpretation of biological information^4^. Regarding currently available Gene Expression Omnibus (GEO) datasets associated with graft restenosis, most studies with uploaded data were on plasma from patients with in-stent restenosis, and three studies were about grafts in animals^5–7^. At this time, no studies about vein graft restenosis in human samples are included.

Herein, clinical samples of occluded vein grafts were collected for transcriptome sequencing and bioinformatics analysis to screen for key genes or proteins affecting the occurrence of vein graft restenosis. We found that ITGB2 was one of the key genes in this process. Silencing of ITGB2 inhibited the proliferation and migration of HVSMCs stimulated by PDGF-BB. Overall, our study demonstrates that ITGB2 is a regulator of HVSMC growth and an essential factor contributing to IH.

## Results

### Identification of DEGs

**Figure 1:**
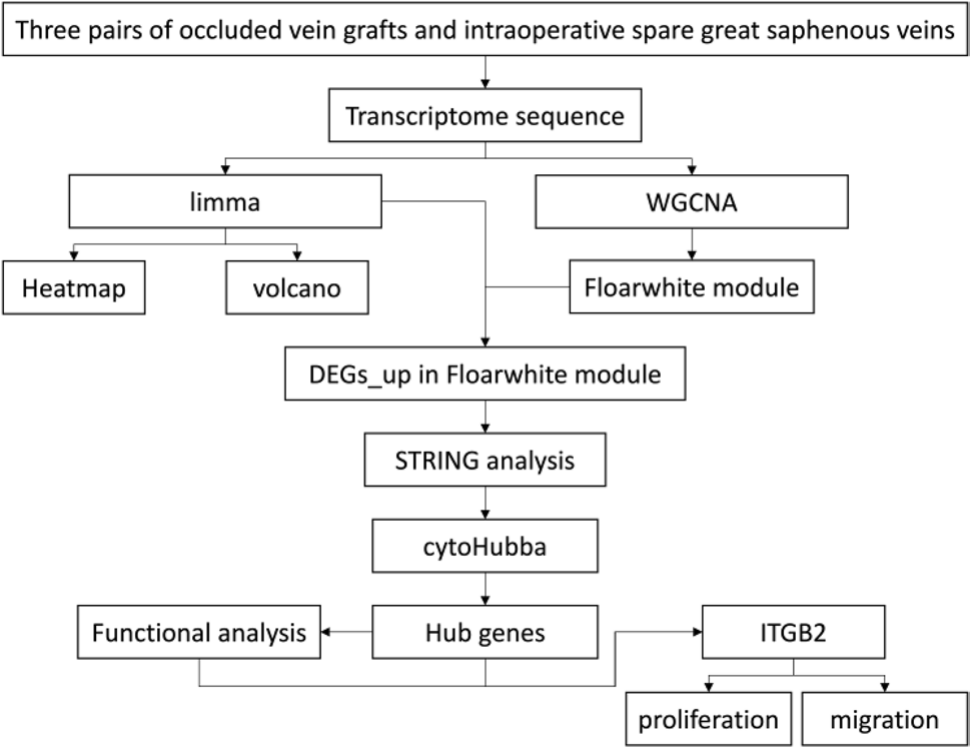
The methodology of the present study.

A flowchart describing the methodology of the present study is shown in Fig. 1. In this study, fifteen patients were included, and more details on their characteristics are shown in Table 1.

**Table 1:** Characteristics of the fifteen redo-CABG patients.

Parameter	Total (n = 15)
Male sex	11 (73.3%)
Hypertension	9 (60%)
Diabetes	2 (13.3%)
Smoking	6 (40%)
Age (years)	67.6 ± 1.158
Postsurgical time (years)	9.267 ± 1.274
Regraft number	2.133 ± 0.1652

**Figure 2:**
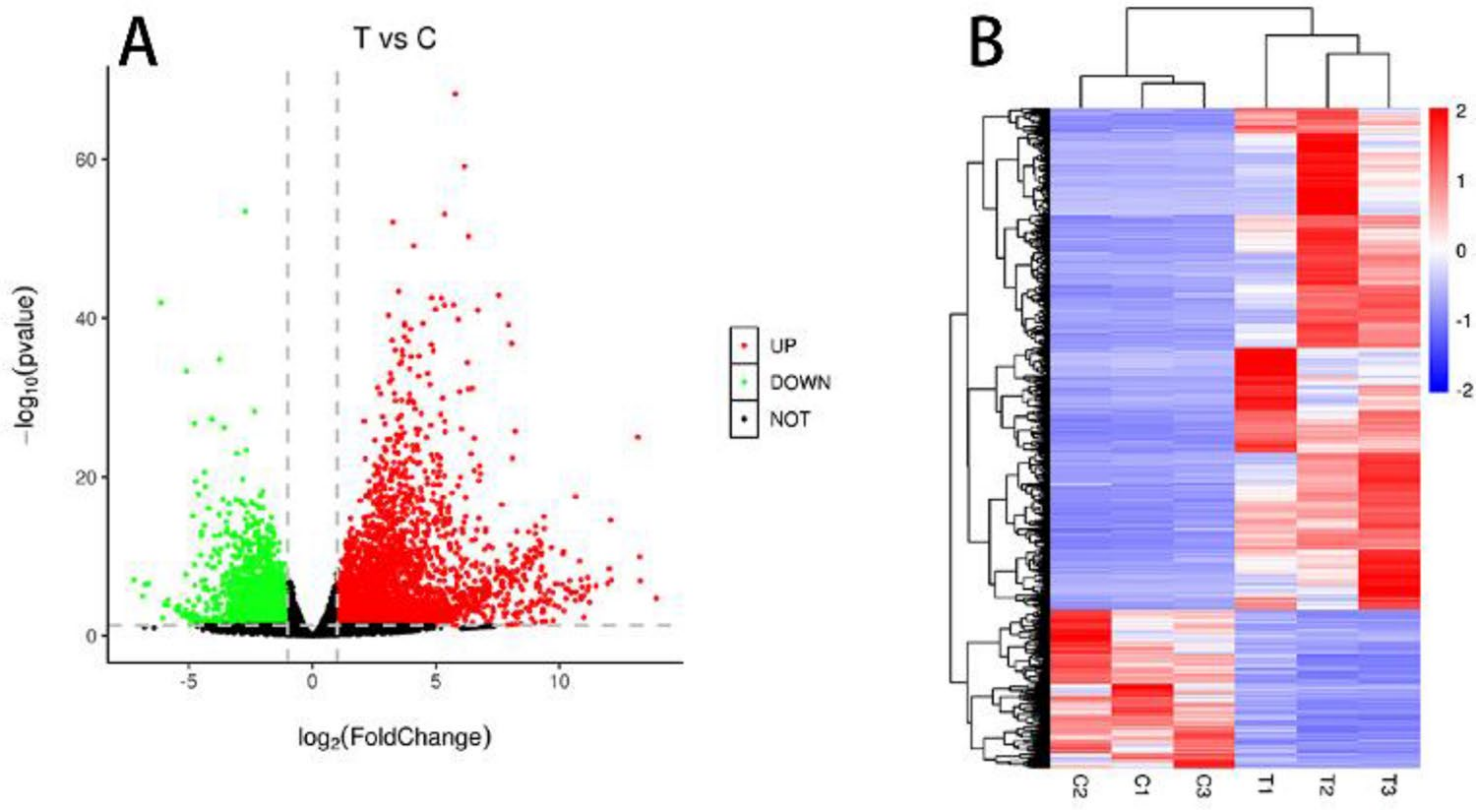
Volcano plot and heatmap of DEGs.

After sequencing, 58,939 genes in our sequencing files were identified. A total of 4479 DEGs in the occluded grafts were identified, namely, 3075 upregulated and 1404 downregulated genes (Fig. 2A,B).

### WGCNA

**Figure 3:**
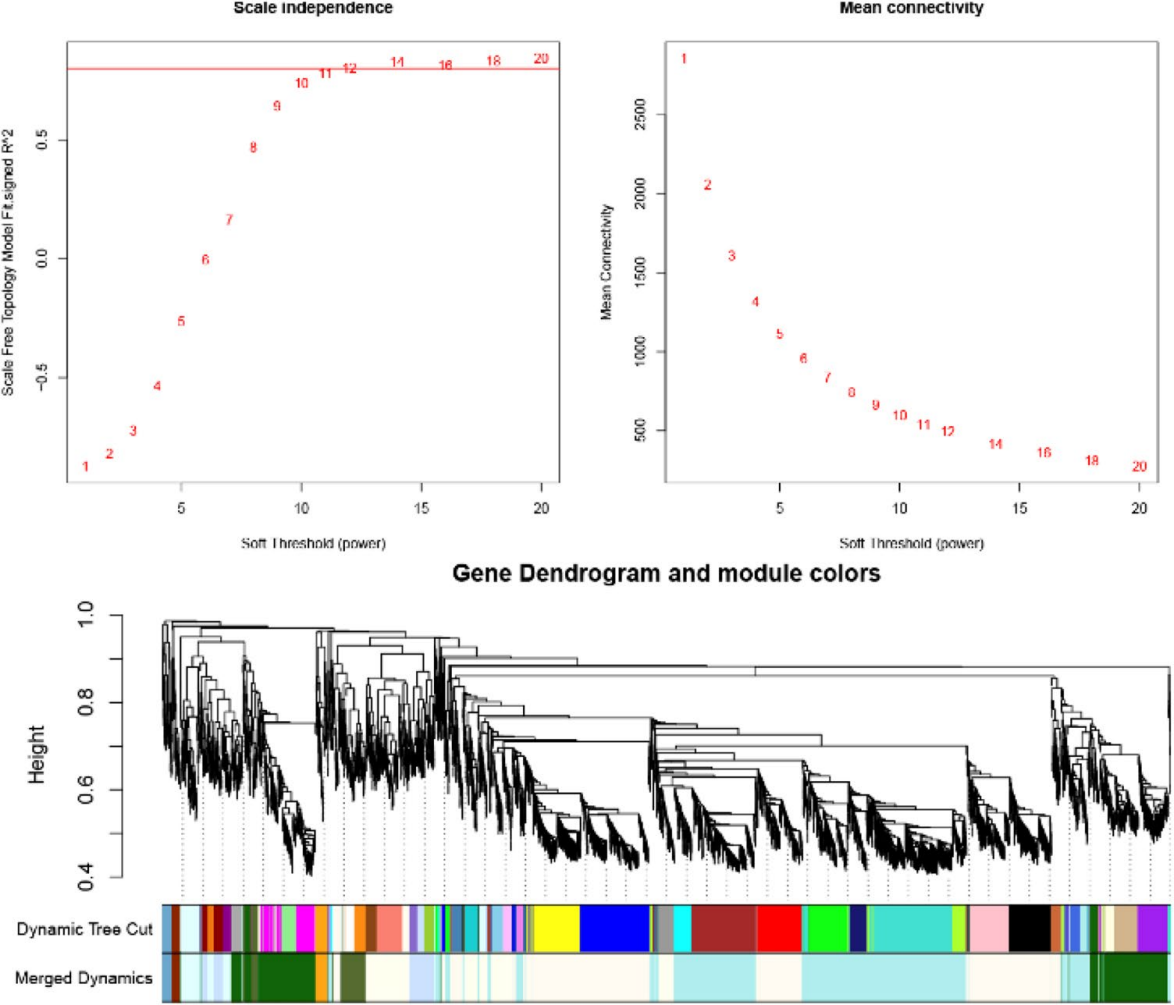
Determination of the soft threshold of the gene coexpression network and module generation based on gene clustering dendrograms. (**A**) Determination of the soft threshold. (**B**) Module generation based on gene clustering dendrograms.

**Figure 4:**
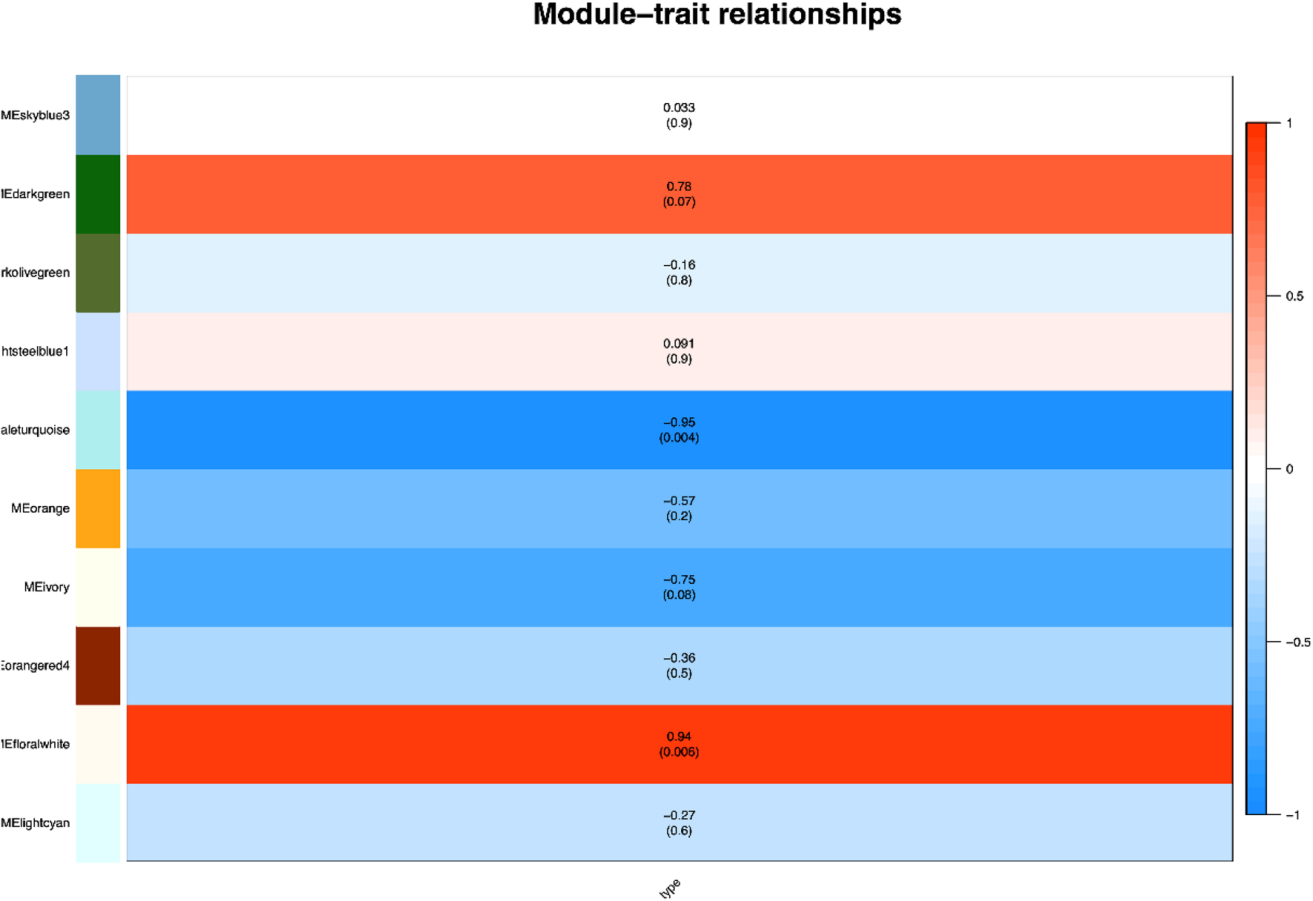
Module-trait associations revealed by Pearson correlation analysis. The colors in the leftmost column indicate different coexpression modules. The numbers in the figure indicate the correlation coefficients between the modules and traits, and the numbers in parentheses are the correlation *P* values.

In our study, WGCNA was conducted with the R package “WGCNA”. Clustering of the patients is shown in Supplemental Fig. 1, where red represents patients with occluded grafts. The key step in WGCNA is the selection of the soft-thresholding power. In our study, the soft-thresholding power was identified by network topology analysis. For WGCNA of vascular restenosis datasets, the soft-thresholding power was 12, and the lowest power of the scale-free topology fitting index was 0.8 (Fig. 3A). A hierarchical clustering tree of all genes in the vascular restenosis database was produced, and 10 important modules were generated (Fig. 3B). Moreover, the dendrogram and heatmap of the genes showed no significant difference in interactions among the modules, demonstrating a high degree of independence between these modules (Supplemental Fig. 2). The floralwhite module had the highest correlation with the of occluded graft status, with a correlation coefficient of 0.94 and a P value of 0.006 (Fig. 4).

### Upregulated DEGs and protein–protein interaction network analysis

The Venn diagram of the DEGs in the floralwhite module and the upregulated DEGs is shown in Supplemental Fig. 3 and contains 615 genes.

**Figure 5:**
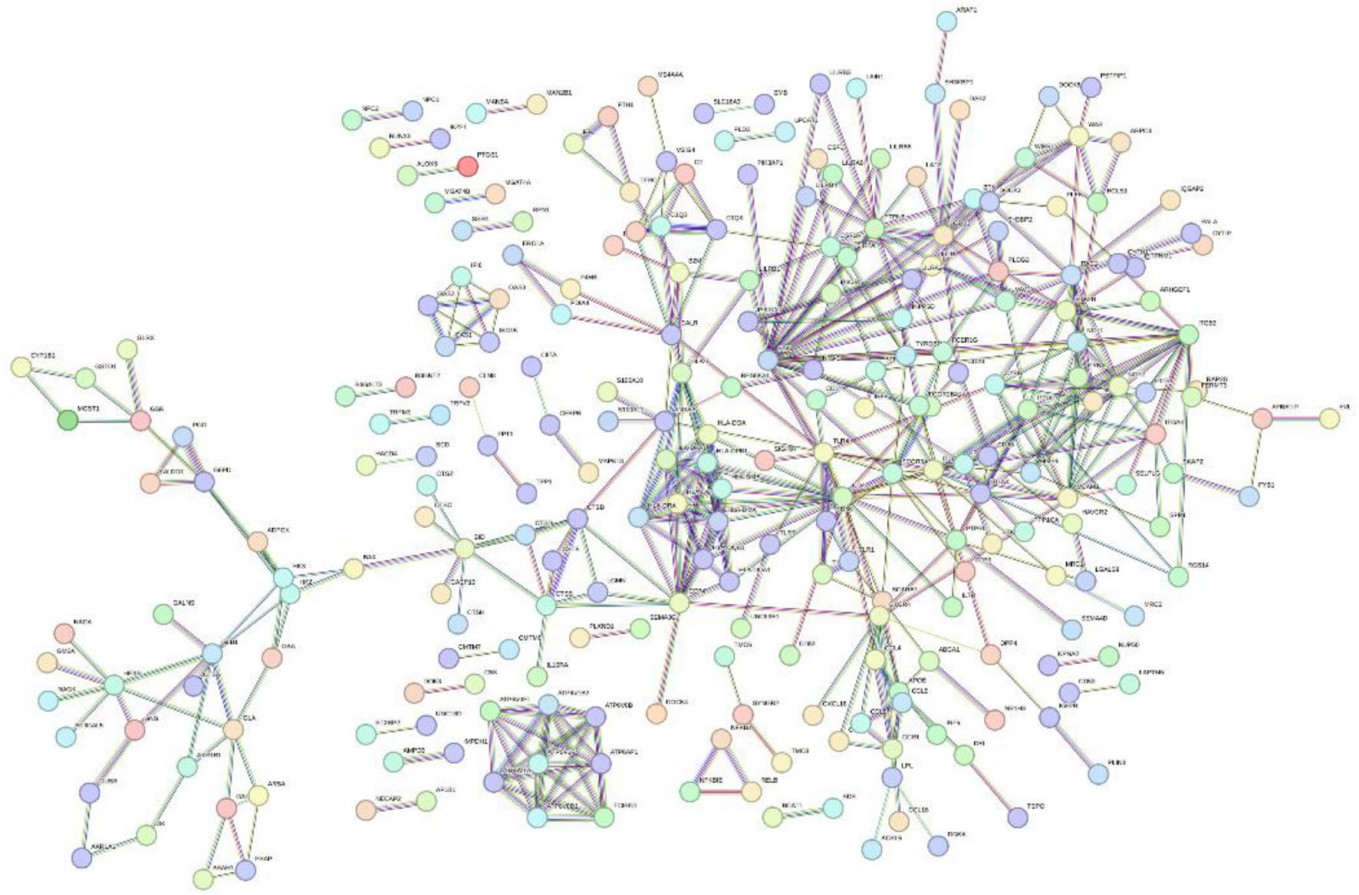
The PPI network of the DEGs_up gene set in the floralwhite module constructed via the STRING online database.

The PPI network of the upregulated DEGs in the floralwhite module was constructed by the STRING online database and visualized by Cytoscape software (Fig. 5). Eleven algorithms were independently used to identify hub genes, and the 6 hub genes identified by the most algorithms were obtained: ITGAM, PTPRC, TLR4, TYROBP, ITGB2 and CD4. A summary of the hub genes is shown in Table 2.

**Table 2:** Top 10 hub genes identified by 11 algorithms.

Algorithm	MCC	DMNC	MNC	Degree	EPC	BottleNeck	EcCentricity	Closeness	Radiality	Betweenness	Stress
1	PTPRC	C3AR1	PTPRC	PTPRC	PTPRC	PTPRC	NRAS	PTPRC	CD4	CD4	CD4
2	ITGAM	HLA-DOA	CD4	CD4	TYROBP	TFRC	ITGAM	CD4	PTPRC	PTPRC	PTPRC
3	CYBB	HLA-DMA	TYROBP	TYROBP	SPI1	ITGB2	PLA2G15	TYROBP	TYROBP	PLEK	PLEK
4	SPI1	MRC1	ITGAM	ITGAM	ITGAM	TYROBP	RAC2	ITGB2	ITGB2	ITGB2	TLR4
5	FCGR2B	MPEG1	SPI1	SPI1	CD86	TLR4	CD86	ITGAM	ITGAM	TYROBP	TYROBP
6	TYROBP	CCR1	ITGB2	ITGB2	ITGB2	TLR7	P4HB	SPI1	SPI1	TLR4	ITGB2
7	FCGR3A	CCL4	CD86	CD86	CD4	HEXB	MARCO	TLR4	TLR4	RAC2	TFRC
8	CSF1R	HLA-DMB	TLR4	TLR4	TLR4	CEBPB	DMXL2	CD86	CD86	TFRC	ITGAM
9	TLR4	FOLR2	PLEK	PLEK	CSF1R	PLEK	CTSB	PLEK	PLEK	CTSD	CSF1R
10	CCR1	LY86	CSF1R	CSF1R	FCGR3A	ITGAM	MAPK13	CSF1R	CSF1R	APOE	RAC2

### Functional and pathway enrichment analyses of the common hub genes

**Figure 6:**
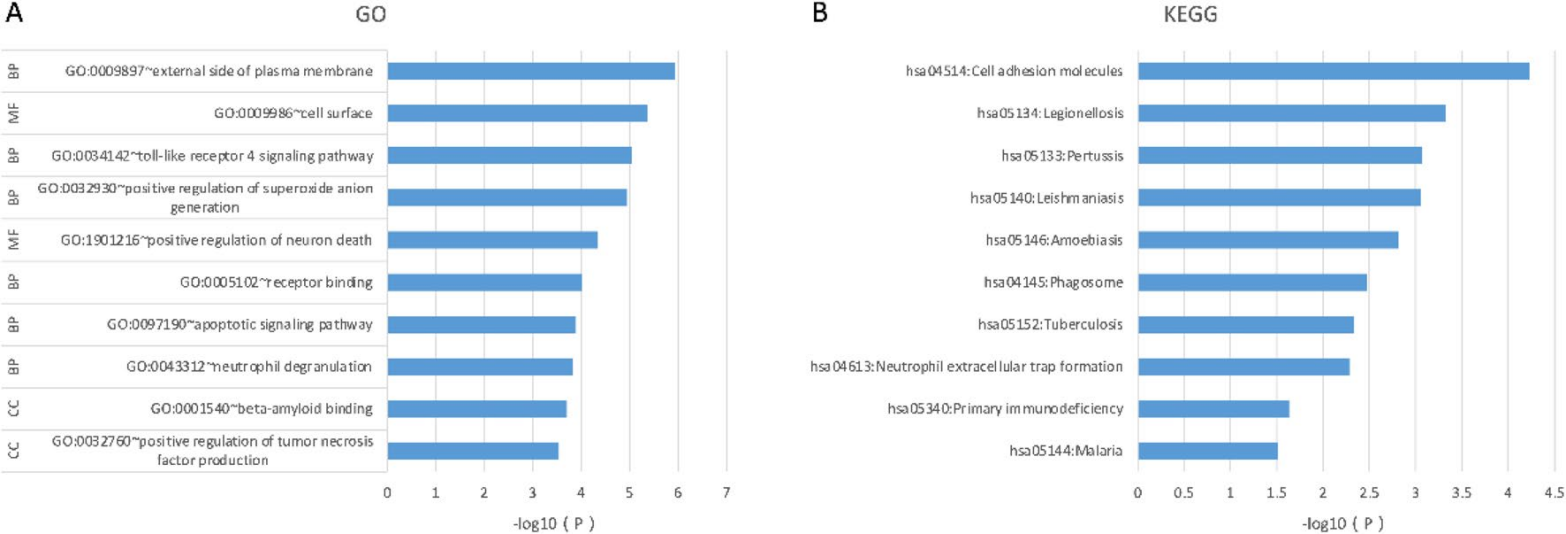
Functional and pathway enrichment analyses of the common hub genes. (**A**) GO analysis of the common hub genes. (**B**) KEGG analysis of the common hub genes.

GO and KEGG analyses of the common hub genes were performed to improve our understanding of the biological functions of these genes. GO analysis of the common hub genes by DAVID showed significant enrichment in components associated with the cell membrane, and the enriched biological processes included neutrophil degranulation and receptor binding, etc. (Fig. 6A). KEGG enrichment analysis showed significant enrichment in the intercellular adhesion pathway, innate immune deficiency pathway and other signaling pathways (Fig. 6B).

### Validation of ITGB2 expression in occluded grafts

**Figure 7:**
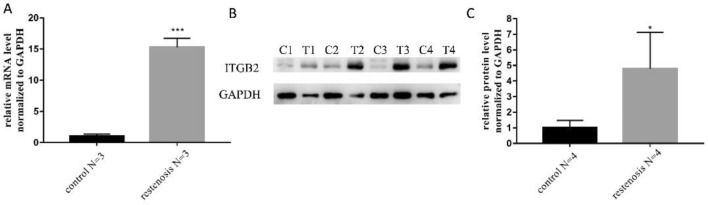
Validation of ITGB2 expression in occluded grafts. (**A**) Relative expression of ITGB2 in occluded vein grafts, as measured by RT-qPCR. (**B**, **C**) Protein expression of ITGB2 in occluded vein grafts, as measured by WB. Control and C group represent the intraoperative spare great saphenous vein samples, and resentosis and T group represent the occluded vein grafts samples. Significance was indicated as *p < 0.05, ***p < 0.001.

Based on the literature and the GO and KEGG analysis results, we finally selected ITGB2 as the target gene, and the expression of ITGB2 in occluded vein grafts and intraoperative spare great saphenous veins was validated by RT-qPCR and WB analyses. The results showed that ITGB2 expression in occluded vein grafts was significantly higher than that in intraoperative spare great saphenous veins (Fig. 7A–C).

### Silencing of ITGB2 significantly decreases HVSMC proliferation and migration stimulated by PDGF-BB

**Figure 8:**
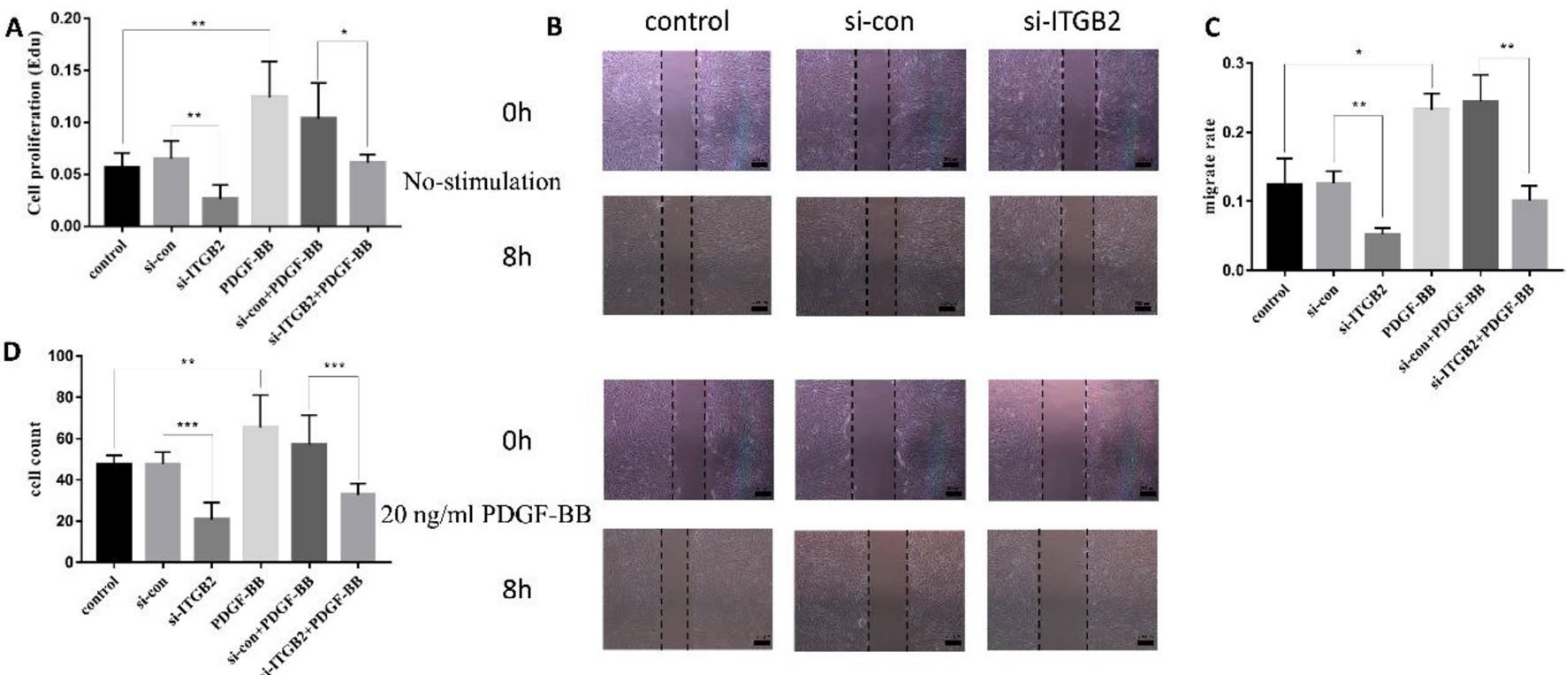
Effect of ITGB2 on HVSMCs stimulated with PDGF-BB. (**A**) EdU incorporation assay to evaluate HVSMC proliferation in the indicated groups 24 h after PDGF-BB stimulation (n = 3). (**B**, **C**) Representative images and migration rates of HVSMCs 0 and 8 h after PDGF-BB stimulation (n = 3). Scale = 100 μm. (**D**) Migration rates of HVSMCs after 24 h of PDGF-BB stimulation. Significance was indicated as *p < 0.05, **p < 0.01, ***p < 0.001.

To study the function of ITGB2 in vessels, primary HVSMCs were generated as previously described and characterized by an immunofluorescence assay (Supplemental Fig. 4). To investigate whether the proliferation and migration functions are regulated by ITGB2, we conducted EdU incorporation, wound healing and transwell assays. First, primary HVSMCs were transduced with si-ITGB2, and the knockdown efficiency was determined (Supplemental Fig. 5). Silencing of ITGB2 significantly decreased cell proliferation by approximately 50% in both the no-stimulation group and PDGF-BB group (Fig. 8A and Supplemental Fig. 6). Likewise, si-ITGB2 inhibited cell migration and invasion compared with that in the si-con group (Fig. 8B–D and Supplemental Fig. 7). Together, these results showed the function of ITGB2 in the proliferation and migration of HVSMCs. Silencing of ITGB2 may inhibit the PDGF-BB-stimulated proliferation and migration of HVSMCs.

## Discussion

Abnormal proliferation of HVSMCs is a major cause of cardiovascular diseases and conditions, such as atherosclerosis, graft restenosis and aneurysm. To our knowledge, no study has assessed graft restenosis with human samples. In the present study, occluded vein grafts and intraoperative spare great saphenous veins were obtained from redo-CABG patients for transcriptome sequencing, and hub genes (ITGAM, PTPRC, TLR4, TYROBP, ITGB2 and CD4) were identified by bioinformatics analysis. GO and KEGG enrichment analyses revealed that the hub genes were associated with cell membrane components, intercellular adhesion and innate immune deficiency.

Atherosclerosis is observed during the late stage of graft restenosis and in the native coronary artery. Atherosclerosis is widely thought to result from abnormal lipid metabolism and chronic inflammation^8^. The ITGAM (also called CR3A, MO1A, CD11B, MAC-1, MAC1A, and SLEB6) gene, located on chromosome 16p11.2, encodes integrin-α M, which is an essential adhesion molecule and might promote the development and progression of abdominal aortic aneurysm by mediating the endothelial cell adhesion and transendothelial migration of circulating monocytes/macrophages^9^. One study showed that CD40L can interact with integrins to elicit monocyte adhesion and migration, representing a novel mechanism of inflammatory signaling in atherosclerosis^10^. TYROBP (also called DAP12, KARAP, PLOSL, and PLOSL1) encodes a transmembrane signaling polypeptide that is a type of transmembrane receptor ubiquitously expressed in macrophages/monocytes, natural killer (NK) cells and neutrophils^8^, and a recent study found that TYROBP promotes atherosclerosis^11^. In recent years, the involvement of NK cells has been documented in various inflammatory responses and early atherosclerosis^12^. Wang et al. revealed that atherosclerotic plaques in APOE mice exhibited high expression of TYROBP^13^. Vein graft restenosis is a chronic process involving atherosclerosis and inflammation. Consistent with these observations, we identified two genes significantly related to these processes.

PTPRC (also known as CD45) is an essential surface protein on hematopoietic and immune cells^14^. PTPRC controls immune function by regulating lymphocyte survival, cytokine responses, and TCR signaling^14^. Deficiency or altered expression of PTPRC is associated with various diseases, including leukemia and lymphoma^15^. PTPRC exerts effects through many mechanisms, including modulating apoptosis and cell survival^14^. In the present study, in silico analysis showed that PTPRC is related to vein graft restenosis, possibly through control of immune function and apoptosis.

Based on the literature, ITGB2 (also known as CD18) was selected as the target gene. The results showed that the expression of ITGB2 in occluded vein grafts was significantly higher than that in intraoperative spare great saphenous veins. An increasing body of evidence suggests that ITGB2 mutation may cause leukocyte adhesion defect type 1 (LAD-1)^16,17^. In recent years, it has been found that ITGB2 mediates a metabolic switch in cancer-associated fibroblasts, promoting oral squamous cell carcinoma proliferation^18^. In a study on myocardial infarction, overexpression of ITGB2 increased the migration and improved the engraftment of adipose-derived stem cells and augmented angiogenesis^19^. Sequencing of carotid atherosclerosis samples showed that ITGB2 was a key gene^20^. The above studies suggest that ITGB2 is tightly associated with the proliferation and migration of cells, in accordance with our results. Next, we found that silencing ITGB2 inhibited the proliferation and migration of HVSMCs.

In summary, our study demonstrates that the key genes related to graft restenosis include ITGAM, PTPRC, TLR4, TYROBP, ITGB2 and CD4. Moreover, ITGB2 knockdown can reduce the proliferation and migration of HVSMCs, a finding that offers novel insights into the prevention of restenosis following CABG.

## Material and methods

### Occluded vein grafts and great saphenous veins

In the present study, a total of fifteen pairs of occluded vein grafts and intraoperative spare great saphenous veins were obtained from patients undergoing clinical redo-CABG; three pairs were used for transcriptome sequencing, and the remaining pairs were used for experimental validation of the hub genes. In addition, the intraoperative spare great saphenous veins of CABG patients were used for primary HVSMC culture. The study was conducted in accordance with the Declaration of Helsinki (as revised in 2013). The study was approved by the ethics committee of Tianjin Chest Hospital (No. IRB-SOP-016(F)-001-02), and informed consent was obtained from all individual participants.

### Identification of DEGs from our sequencing data files

After transcriptome sequencing, we obtained the transcriptome expression profiles of three pairs of vessels. The expression profiles were normalized with the R package “limma”. The differentially expressed genes (DEGs) were screened by the R package “limma” based on the cutoff criteria of |log_2_FC|> 1 and *P* value < 0.05.

### Weighted gene co-expression network analysis (WGCNA)

WGCNA was performed with the R package “WGCNA” (https://cran.r-project.org/web/packages/WGCNA/index.html)^21,22^. First, a co-expression network containing all genes was constructed, and the 20% of genes with the highest variance were used for further analysis. Samples were used to construct the adjacency matrix. Then, the adjacency matrix was transformed into a topological overlap matrix (TOM). Genes were classified into different modules by measurement of differences based on the TOM. In this study, we set the minimal gene module size as 30 and the threshold to merge similar modules as 0.25 to explore modules significantly correlated with clinical traits.

### Identification of upregulated DEGs in the floralwhite module and construction of protein–protein interaction (PPI) networks

First, a Venn diagram was used to determine the intersection of the set of genes in the module with the highest correlation with clinical traits and the set of upregulated DEGs to identify upregulated DEGs in the floralwhite module; this was performed with FunRich, a biological analysis software (http://www.funrich.org/). The STRING database (http://string-db.org/^23^ is widely recognized to collect, store and integrate publicly available sources of protein–protein interaction information and supplement these sources through computational predictions. In this study, PPI networks of the upregulated DEGs in the floralwhite module were analyzed through the STRING database (confidence score > 0.9)^23^. Subsequently, the PPI networks were visualized and analyzed with Cytoscape software and the cytoHubba plugin. All algorithms were applied to screen for hub genes. The top 6 genes were regarded as hub genes.

### Functional annotation of the hub genes

The Gene Ontology (GO) database is a comprehensive resource of calculable knowledge about the functions of genes that is widely used by the biomedical research community to analyze omics and related data^24^. The Kyoto Encyclopedia of Genes and Genomes (KEGG) database has been reported to link genomic information with higher-order functional information^25^. In addition, the Database for Annotation, Visualization and Integrated Discovery (DAVID, https://david.ncifcrf.gov/) provides a comprehensive set of functional annotation tools for investigators to understand the biological meaning behind large lists of genes^26^. In our study, GO and KEGG analyses of the hub genes were performed with the online DAVID tool (genes with the 10 highest −log_10_ *p* values).

### Real-time quantitative PCR (RT-qPCR)

Total RNA was isolated from the occluded vein grafts and intraoperative spare great saphenous veins of patients using TRIzol reagent (Invitrogen, Carlsbad, CA, USA) according to the manufacturer’s protocol. Total RNA from primary HVSMCs was isolated using a Takara reagent according to the manufacturer’s protocol (Takara, Shiga, Japan). Complementary DNA (cDNA) was reverse transcribed from 2 μg of RNA by using the Takara reverse transcription system (Takara), and real-time PCR was performed with SYBR Green mix on the 7500 real-time PCR system (Applied Biosystems; ABI, Waltham, Ma, USA). ITGB2 expression was normalized to glyceraldehyde 3-phosphate dehydrogenase (GAPDH) expression, and relative expression levels were calculated with the 2-^ΔΔCT^ method.

The primer sequences were as follows: ITGB2 forward: 5′-GAGTGCCTGAAGTTCGAAAAG-3′, reverse 5′- TCATCCACATAGATGAGGTAGC-3′; GAPDH forward: 5′- AAAAGCATCACCCGGAGGAGAA-3′, reverse 5′- AAGGAAATGAATGGGCAGCCG-3′.

### Western blot (WB) analysis

Proteins from occluded vein grafts and intraoperative spare great saphenous veins of patients were extracted following the kit instructions (Solarbio, Beijing, China). Then, the proteins were separated by SDS‒PAGE and electrophoretically transferred to PVDF membranes (Millipore, Burlington, MA, USA), which were incubated with gentle shaking overnight at 4 °C with a primary antibody against ITGB2 (Santa Cruz, TX, USA) or GAPDH (Proteintech, Wuhan, China) and were then incubated with horseradish peroxidase-conjugated secondary antibodies for 1 h at room temperature (Proteintech). Bands were visualized with ECL reagents (Thermo Fisher Scientific, Waltham, MA, USA).

### Primary HVSMC culture and identification

As previously described^27^, HVSMCs were isolated from intraoperative spare great saphenous vein segments obtained from patients undergoing CABG. For cell culture, HVSMC medium (ScienCell, USA) supplemented with 20% fetal bovine serum (FBS, Gibco, USA), 1% growth factors (ScienCell), and 1% penicillin/streptomycin (Gibco) was used. HVSMCs were characterized by immunofluorescence staining for smooth muscle-specific α-SMA. All experiments employed HVSMCs at passages 3–5.

### siRNA transfection and knockdown efficiency of si-ITGB2

Primary HVSMCs were transfected with a specific small interfering RNA (siRNA) or control siRNA using Lipo3000 transfection reagent (Thermo Fisher Scientific, China) following the manufacturer’s instructions. The siRNA targeting ITGB2 was purchased from Santa Cruz Biotechnology (TX, USA). The knockdown efficiency was verified 24 h post-transfection by RT‒qPCR and WB analyses. After 24–48 h of transfection, HVSMCs were used for subsequent experiments. For PDGF-BB treatment, after 24 h of transfection, HVSMCs were serum-starved for 12 h before incubation with 20 ng/ml PDGF-BB for 24 h.

### EdU incorporation assay

The EdU incorporation assay was conducted using the BeyoClick™ EdU Cell Proliferation Kit with Alexa Fluor 594 (Beyotime, China). After transfection for 24 h and serum starvation for 12 h, HVSMCs were washed with PBS. Then, medium supplemented with or without 20 ng/ml PDGF-BB was added to the wells. After 24 h of incubation, half of the medium was kept, and EdU was added to the medium to maintain a concentration of 10 μM per well. The HVSMCs were incubated for 2 h at 37 °C in a 5% CO_2_ atmosphere. After incubation, the HVSMCs were washed with PBS and fixed with 4% paraformaldehyde at room temperature for 15 min before being stained with DAPI for 10 min. After an additional wash in PBS, the cells were observed under an inverted microscope.

### Wound healing assay

After 48 h of transfection, HVSMCs were serum-starved for 12 h. A linear wound was made with a 200 μl pipette tip in the middle of each well of a six-well plate. After washing with PBS, 2 ml of serum-free medium with or without 20 ng/ml PDGF-BB was added, and continuous imaging was initiated. This time was recorded as 0 h. After 8 h, images were acquired continuously in each group. The differences between each group at 0 h and 8 h were quantified using ImageJ software, and the rate of wound healing was calculated using the following formula: Wound healing rate = ((area of scratch at 0 h –area of scratch at 8 h)/area of scratch at 0 h) × 100%. Each experiment was repeated three times.

### Transwell migration assay

Transwell chambers (24-well Transwell chambers, Corning Inc., NY, USA) were used for the migration assay. After transfection for 48 h, HVSMCs were resuspended in serum-free medium, and the cell density was adjusted to 2 × 10^4^ cells/ml. Moreover, 200 µL of the cell suspension was seeded into the upper chambers. The lower chambers contained 600 µL of 10% FBS medium with or without 20 ng/ml PDGF-BB. Following 24 h of incubation, the cells that invaded into the lower surface of the membrane were fixed with 4% paraformaldehyde, stained with 0.1% crystal violet, and counted in five random fields under a bright field microscope. Each experiment was repeated three times.

### Statistical analysis

All data are expressed as the means ± SEMs, and all statistical analyses were performed with GraphPad Prism 7.0 software using Student’s unpaired t test when comparing the two groups. A *P* value of < 0.05 was considered to indicate statistical significance.

### Supplementary Information

Supplementary Figures.

## Data Availability

The datasets generated and analysed during the current study are available in the Gene Expression Omnibus (GEO) repository, GSE241205.
