# Supplementary material for: Identification of important genes related to HVSMC proliferation and migration in graft restenosis based on WGCNA
Source: Sci Rep. 2024 Jan 12;14:1237. doi: 10.1038/s41598-024-51564-z (PMC10786872; doi:10.1038/s41598-024-51564-z)
Supplement: Supplementary file 1 — Supplementary Figures. [file 41598_2024_51564_MOESM1_ESM.pdf]

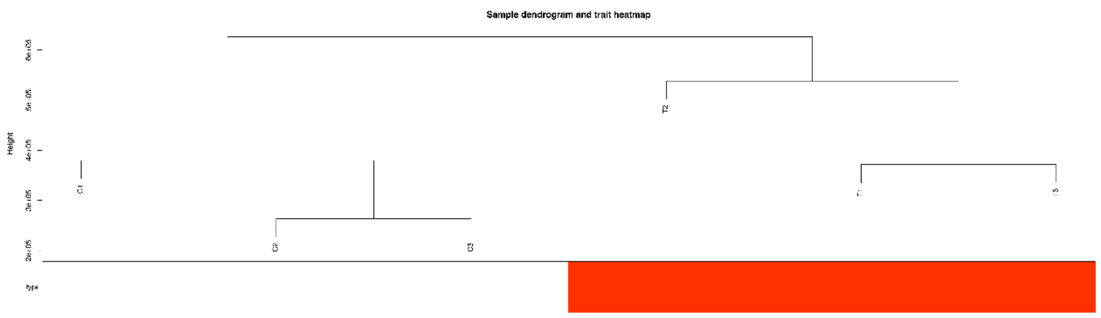

Supplemental Fig 1 The cluster of the vessel samples from redo-CABG patients

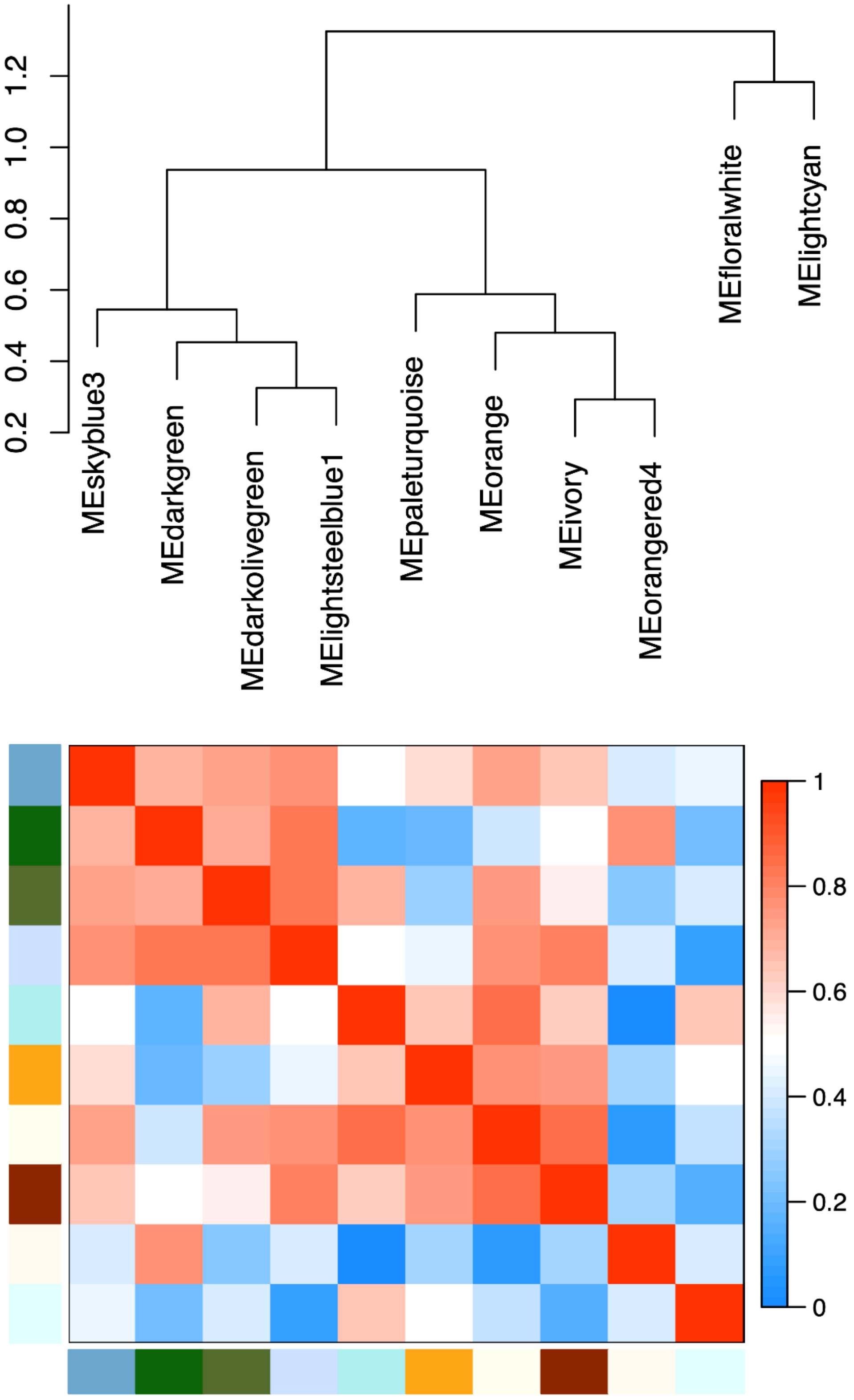

Supplemental Fig 2 The dendrogram and heatmap of genes in different modules

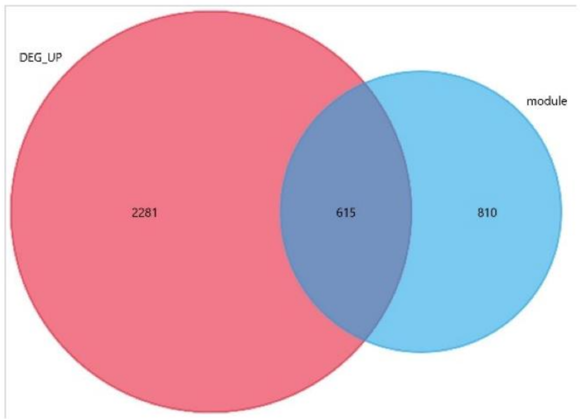

Supplemental Fig 3 The Venn plot of DEGs in the floralwhite model and upregulated DEGs

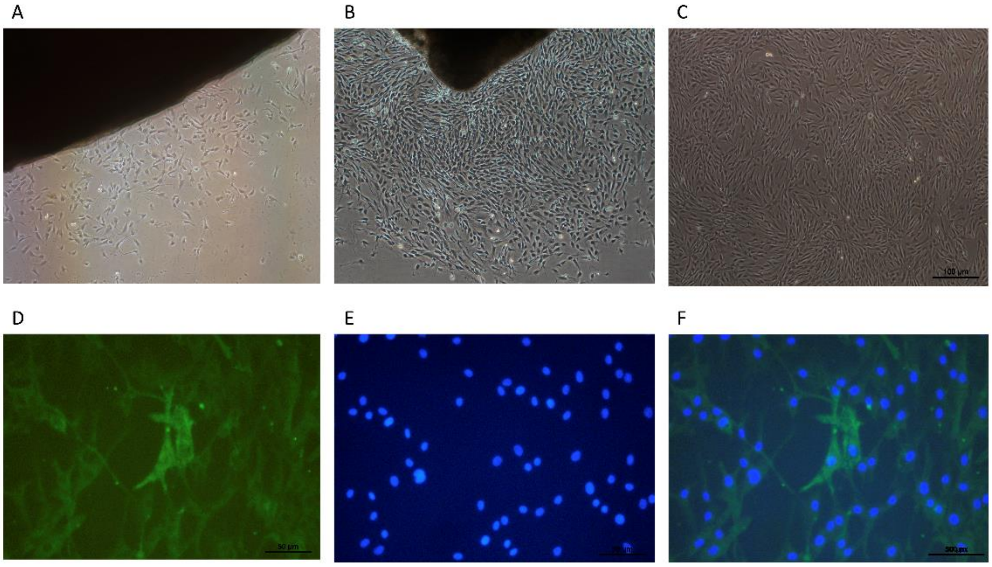

Supplemental Fig 4 The primary HVSMC was cultured by tissue-patch method and identified by an immunofluorescence assay

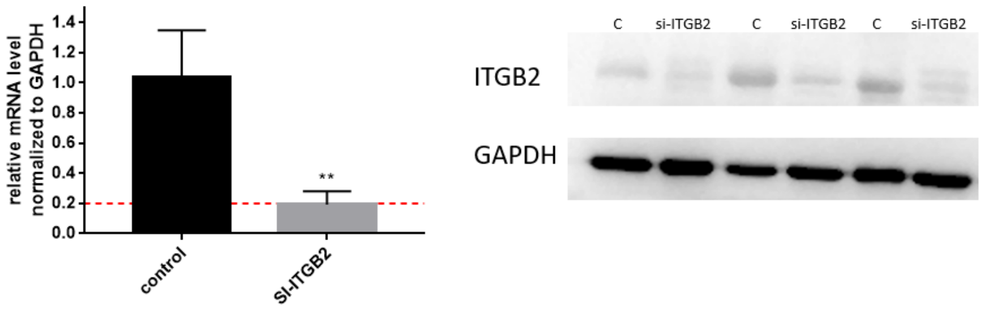

Supplemental Fig 5 The knockdown efficiency of ITGB2 was calculated by RT-PCR and WB

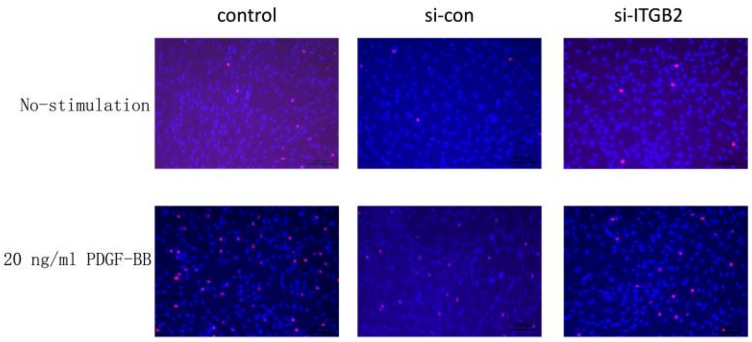

Supplemental Fig 6 Effect of ITGB2 on HVSMCs stimulated with PDGF-BB. Typical images of EdU incorporation assay to evaluate HVSMC proliferation in the indicated groups 24 h after PDGF-BB stimulation (n=3).

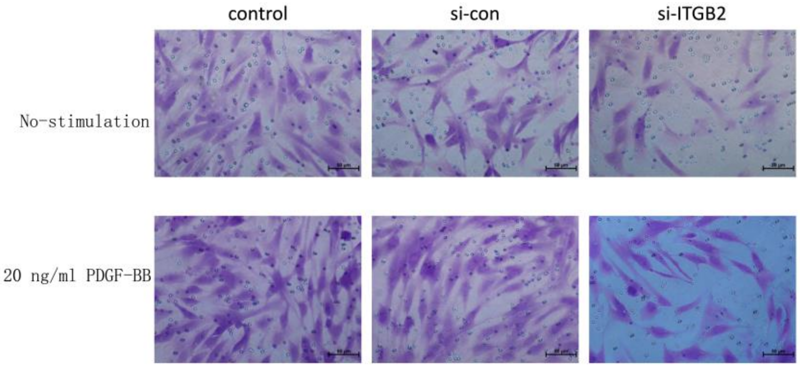

Supplemental Fig 7 Effect of ITGB2 on HVSMCs stimulated with PDGF-BB. Typical images and migration rates of HVSMC after 24 h stimulation of PDGF-BB.

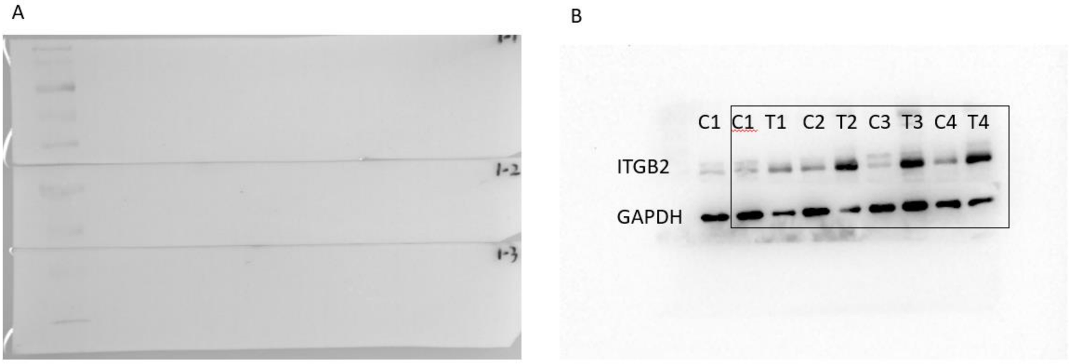

Supplemental Fig 8 The blots of ITGB2 expression validations by WB were from the same gel, and under the same exposure conditions. A. Full-length images of Fig 7 B in the white conditions. B. The bolts were cut prior to hybridisation with antibodies. The middle piece was not shown in the coloration step. The part in the black rectangle was shown in Fig 7B.

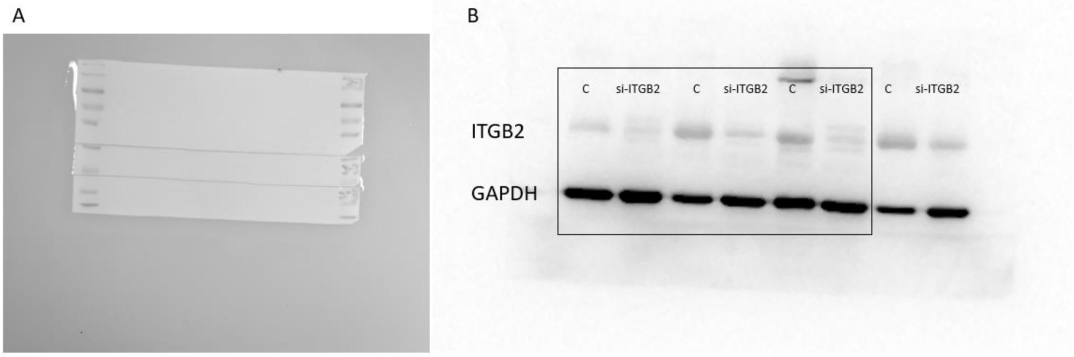

Supplemental Fig 9 The blots from WB for calculating the knockdown efficiency were form the same gel, and under the same exposure conditions. A. Full-length images of Supplemental Fig 5 in the white conditions. B. The blots were cut prior to hybirdiastion with antibodies. The middle piece was not shown in the coloration step. The part in the black rectangle was shown in Supplemental Fig 5.
